# Your Breath

**Published:** 1905-02-15

**Authors:** R. B. Tuller


### YOUR BREATH.

BY R. B. TULLER, D.D.S.

(Reprinted from January "American Journal.")

I'm a
Friend of yours and
I want to have a heart to heart talk with you; yes, you.

You are a pretty decent-looking fellow. Your clothes look reasonably clean and tidy, and your linen fresh; and your hands look soft and well-cared for, and your nails are well manicured—not professionally manicured, perhaps, but a very decent job, if you did do it yourself.

The barber seems to have had you in hand, too; even to a facial massage with plenty of hot towels.

I don't wish to offend you in the least—not for anything; and, say, now, you must not be offended. Somebody has *got* to tell you—if you haven't a wife—and sometimes if you have.

But please don't look me in the eye. Kindly turn your face away while I propound a question.

Do you make Limburger cheese a part of your daily diet—no? Well, it might be an improvement if you did. Aha! are you on?

I've been trying to approach this matter as gently and delicately as I could, and I'm glad you've caught on. It is *rank*. You may have your opinion about my eating Limburger, but this is my inning, not yours. I have the floor and I'm talking to you.

I just wanted to tell you what you probably don't know about yourself. Your breath——

"Oh, the smell of the jessamine-flower" is much more agreeable; and even a burnt woolen rag—well, you can guess what this is, anyway.

To be plain, the afflatus of that embrasure of your physiognomy is not of that divine inspirational character that impels one to linger in the aroma (?) and think lofty thoughts and sentiments, any more than it would to loll on a hot summer day on the banks of "Bubbly Creek," in which the offal of the Chicago stock yards discharges and lies festering for many a day. No, I don't think.

On the contrary, cuss words float through one's brain, and not infrequently, audibly through the air; and the first impulse (and the last), is to flee—flee as a bird.

But what about those patients of yours? They, no doubt, think cuss words all right, and the sin is on your head; but they can't flee—not for the moment; but they may at the first opportunity, and never return.

Now, sir, this is where I'm your friend. I'm telling you in this quiet, *sub-rosa* way so that you may try and correct the evil and avert any such catastrophe. That is what it is, and a dum poor advertisement when your patient's tongue never forgets to give you a left-hander after this fashion: "what a pity it is that such a nice, pleasant gentleman and fine operator has such a terrible breath! Wuh!"

You are not the man? What? Do you deny the *rank* impeachment? Well, sure, there is no suggestion of peach or mint about it. It is *vile*.

Oh, possibly not to-day, because your health is better than yesterday—your stomach, anyway—and because you have taken extra precautions; but tomorrow, or next day, possibly in an hour after taking some kinds of food. You can never tell, yourself, whether your breath is tainted or not, so don't get sore at me for giving you the tip and being plain about it. Oh, yes, of course, if you have a very dark brown taste in your mouth you may know that your breath is not like an infant's.

You say you take every precaution as regards cleanliness and hygiene? You brush your teeth and use antiseptic washes three or four times a day? Good! Keep it up! But do you make any effort to regulate your diet—except possibly to deny yourself onions and garlic? Is your digestion good? Knowing it is not, do you take any precautions against the putrifying odors that are extremely evident?

Do you drink? Excuse me, I did not ask you to have anything. Not me. If any one asks you, begin the new year right and politely but firmly decline.

Let your motto be,

No "suds" for me.

Isn't it strange what drawbacks there are to a number of good things in this world—and you can't disguise them, not not even with cloves or cardamoms. No, the only thing to do is to let it alone. Cling to the sprinkler, though the spray takes all the crease out of your trousers.

But you smoke? Oh, yes, I've seen you. I've seen you (some of you) with one of those *nasty* cigarettes in your mouth, and here in a civilized community. What's that? You've seen me with rope? Well, not from any craving inclination to smoke rope. Mine is a two-for-fifty taste, with two-for-five resources. But *you* are the target of this topic, not I. Don't go out and smoke anything and go back and breathe it into the sensitive face of the patient you've got down in the chair and who can't get away. Remember, that at your sweetest you do not smell always like the delicate odor of attar of roses.

Oh, of course, you rinse your mouth and even scrub out your mustache, but tobacco takes several hours to fade away, and you know it. Such efforts as you may use to modify or suppress the disagreeableness is the least you can do, but why don't you use some horse sense? Horses do not pollute their mouths with tobacco.

When the poet was stirred by the real divine afflatus to pen the *beautiful* lines below, he no doubt had been stirred many a time before by the rank and rancid afflatus of a tobacco mouth. Wuh! Here are the beautiful lines. Commit them to memory and follow the resolution of little Robert:

"I'll never use tobacco, no;
For 'tis a filthy weed;
I'll never put it in my mouth,"
Said little Robert Reed.

It is a long time since I saw the poem and I may not have quoted it verbatim. In fact, I've been questioning in my own mind if the boy's name was not Jimmie instead of Robert, and Reed may be spelled wrong. Any way, the sentiment of the thing is there. Inhale that instead of tobacco—and especially cigarettes.

We have to inflict woes enough on our patients without overwhelming them with a rank breath. Sometimes they come back at us with a worse one, it is true; but we have remedies at hand for temporary relief, while they have none—except to get away from us as soon as they can.

The trouble is, as I have already said, that not one person in a thousand suspects that he has a bad breath until told by some good friend.

Now, what is there to be done for ourselves?—I mean for you. I forgot I was talking just to *you*.

Well, use every precaution to keep your stomach in a first-class normal condition, and avoid any ill-smelling diet—that is, that is ill-smelling after it is down. Yes, you have got to be mighty careful of the things you may eat and drink, if you want a nice, clean breath, besides using all hygienic measures (and use them often) for odors that emanate from fermenting and decaying food particles in the mouth. Make it a point to use a good deodorant and antiseptic mouth-wash before beginning service for each patient; and if the service is at all protracted, repeat the dose once, twice or thrice. Treat yourself as a suspect at all times and use precautions, and keep your mouth closed while operating. The older you grow the more the need. You know it well enough.

Dioxogen is my favorite mouth-wash, or something of that order. Nothing burrows into the deep and obscure recesses and brings out the offensive substances better than some of the $H_2O_2$ preparations of about 3 percent strength. When I get hold of a patient with an offensive breath I very soon introduce a dose of peroxide into the mouth on some pretext or other, or without any, and then follow with peppermint water or sanitol.

When we come to nasal catarrhal conditions in ourselves and others, then we have sometimes got something hard to contend with, and should refer and urge the patient or ourselves, may be, to consult a physician. Disorders of the stomach, too, should generally go to the physician.

You still think I am talking to some one else, and that *you* are all right? Well, what you think you are and what you are, are very different propositions very often. Get wise early in the game and whatever you are, try and not be a stingy fellow in the effort to keep your breath as pure and sweet as a new-born babe's or a nice, tidy girl of sweet sixteen.

You know and I know some dentists who set us to wondering how on earth they hold a practice, but if we had something like litmus to test for bad smells in our mouths a good many of us might get a shock. Don't take any chances.

Anyway—

Reek not thy breath with brew or still,
And 'gainst tobacco set thy will.
Do not distend thy stomach out
With onion-salads and sourkraut.

